# Hair Growth Promotion Effect of Nelumbinis Semen Extract with High Antioxidant Activity

**DOI:** 10.1155/2021/6661373

**Published:** 2021-03-13

**Authors:** Hyeon Ju Park, Guang-Ri Jin, Jae Hyun Jung, Su Bin Hwang, Su Hyun Lee, Bog-Hieu Lee

**Affiliations:** Department of Food and Nutrition, Chung-Ang University, Anseong, Gyeonggi-do 17546, Republic of Korea

## Abstract

This study investigated the hair regeneration promotion and hair loss prevention properties of Nelumbinis Semen (NS) extract *in vitro* and *in vivo*. The effect of NS on the proliferation and migration of human dermal papilla cells (hDPCs) was measured *in vitro* via CCK-8 and scratch migration assays, after which the antioxidant activity of NS was also quantified. NS extracts were then applied to the back of 7-week-old C57BL/6 mice for 3 weeks to monitor hair growth patterns and hair follicle (HF) histology. The mice were divided into three groups: negative control group (NC; DMSO), positive control group (PC; 3% minoxidil), and experimental group (NS extract 1,000 ppm). Moreover, to study the molecular mechanisms by which NS extract regenerates hair growth, real-time PCR was used to analyze factors related to the hair growth cycle. The NS extracts were found to possess high antioxidant properties due to their high flavonoid contents and electron-donating ability. Moreover, NS extracts enhanced hDPC proliferation and migration in a concentration-dependent manner (15.63–125 ppm). The hair growth index and growth area of the NS group (2.81 score, 81%) on day 14 were higher than those of the PC group (2.65 score, 68%) (*p* < 0.05). Additionally, the HFs of the NS group were located deep in the subcutis, similar to the PC group with developed hair roots. Moreover, the mRNA expression of VEGF and IGF-1 was higher in the NS group compared to the PC group, whereas TGF-*β*1 expression was lower (*p* < 0.05). Our findings indicate that NS modulates hair growth by increasing IGF-1 and VEGF expression while inhibiting that of TGF-*β*1. Therefore, our findings suggest that NS extract is a promising new hair loss treatment derived from a natural substance that helps promote hair growth and prevent hair loss.

## 1. Introduction

Alopecia is a disease characterized by progressive hair loss from the scalp and other areas of the body. Recently, there has been a rapid rise in population-wide hair loss cases. This is due to genetic factors, as well as to increased stress, dietary changes, and hormone-related problems [1, 2]. According to the Korea National Health Insurance Service's morbidity statistics, the number of patients treated for alopecia areata increased by 24.9% from 136,248 (male: 69,280; female: 66,968) in 2010 to 170,149 (male: 92,759; female: 77,390) in 2018 [3]. The types of alopecia were largely classified into the following categories: androgenetic alopecia (AGA), female pattern hair loss (FPHL), alopecia areata (AA), postpartum telogen effluvium, telogen effluvium, and traction alopecia [4].

Currently, finasteride and minoxidil are the only drugs approved by the Food and Drug Administration (FDA) for the prevention of hair loss and promotion of hair growth. Finasteride is known to improve AGA by inhibiting the activity of 5*α*-reductase II;, thereby inhibiting the conversion of testosterone (T) to dihydrotestosterone (DHT). On the other hand, minoxidil is a vasodilator that enhances hair growth by inhibiting hDPC apoptosis [5, 6]. However, these FDA-approved drugs have been linked to several side effects including erectile dysfunction, infertility, and allergic dermatitis. Therefore, research and development of novel therapeutic agents capable of preventing alopecia and enhancing hair growth are necessary. Moreover, these efforts should particularly focus on natural products, which may result in fewer or less severe side effects.

hDPCs, which are involved in the hair growth cycle, are located at the bottom of the HFs and are wrapped by hair matrix cells. Hair growth can be regulated by controlling the proliferation, division, and apoptosis of hDPCs through the activity of growth and inhibitory factors such as insulin-like growth factor-1 (IGF-1), vascular endothelial growth factor (VEGF), and transforming growth factor-*β*1 (TGF-*β*1). IGF-1 is considered a representative hair growth factor that promotes epithelial cell proliferation and significantly increases the length of HF tissues [7]. Importantly, this factor also inhibits anagen to catagen transition [8]. VEGF is another factor that induces growth and differentiation of hair root cells by both improving blood circulation and enhancing vascular endothelium growth [8, 9]. Therefore, VEGF prevents hair loss by increasing HF size and hair thickness [10, 11]. Conversely, TGF-*β*1 causes apoptosis of hair follicle matrix cells and alopecia by rapidly entering the catagen phase. Additionally, incomplete hair follicle regeneration leads to anagen to catagen transition and reduces HF sizes, resulting in thin and short hair [12, 13].

Nelumbinis Semen was dried by removing the pericarp of ripe lotus seeds. In China, NS has long been regarded (i.e., since ancient times) as an effective therapeutic agent due to its beneficial effects on the spleen, kidney, heart, and eyes [14]. According to a recent study, NS is rich in proteins, minerals, and unsaturated fatty acids, as well as various bioactive compounds that enhance human health such as alkaloids and flavonoids. Therefore, NS is widely used in the food and medicine industries [14, 15]. NS has been mainly used to treat neurological disorders, insomnia, and postmenstrual depression in women and has been reported to have antiviral properties, as well as a liver protectant, antioxidant, memory-enhancing, and anti-inflammatory effects [16–18].

Oxidative stress is among the leading causes of hair loss [19]. Previous studies have demonstrated that naturally occurring plant antioxidants can reduce oxidative stress [20], and NS has been found to possess potent antioxidant properties [14, 15]. Therefore, we hypothesized that NS would effectively promote hair regeneration and prevent hair loss. Several studies are currently assessing the nutritional properties of NS extract; however, no previous studies have systematically characterized the effect of NS extract on the hair growth cycle. Therefore, our study sought to (1) assess the effect of NS extract on hDPCs proliferation and migration *in vitro* and (2) characterize the hair growth-promoting properties of NS *in vivo* using C57BL/6 mice. Additionally, the molecular mechanisms by which NS extract induces hair regeneration were assessed by measuring the expression of various hair growth-associated cytokines.

## 2. Materials and Methods

### 2.1. Preparation of NS Extracts

NS extract was purchased from Korea Plant Extracts at the Korea Research Institute of Bioscience and Biotechnology (Daejeon, Korea). Methyl alcohol (50%) was used as the extraction solvent of the sample, which was then dissolved in dimethyl sulfoxide (DMSO).

### 2.2. Antioxidant Activity of NS Extracts

Total polyphenol content was determined using Folin-Ciocalteu's method [21]. Briefly, 40 *μ*l of NS extract prepared with 50% methanol at a concentration of 0.2 mg/ml was mixed with 50 *μ*l of Folin-Ciocalteu's phenol, which was then added to 160 *μ*l of 10% sodium carbonate solution after 3 min at room temperature. The mixture was kept at room temperature for 30 min, after which absorbance was measured at 700 nm using a 7315 UV spectrophotometer (JENWAY, Staffordshire, United Kingdom). Total polyphenol content was expressed as mg of gallic acid equivalent (GAE)/g.

To determine the total flavonoid content, 0.5 ml of the 0.2 mg/ml dissolved in ethanolic NS extract was mixed with 5 ml of diethylene glycol (Duksan Pure Chemicals, Ansan, Korea). After 5 min at room temperature, the mixture was added to 0.5 ml of 1N NaOH (Duksan Pure Chemicals, Ansan, Korea) and incubated for 1 hour in a 37*°*C water bath. Absorbance was measured at 420 nm using a 7315 UV spectrophotometer (JENWAY, Staffordshire, United Kingdom). Total flavonoid content was expressed as mg of naringin (Tokyo Kasei Kogyo Co., Ltd., Tokyo, Japan) equivalent (NE)/g.

The DPPH scavenging activity of NS extract was assessed as described by Blois [22], with some modifications. Briefly, 0.5 ml of NS extract diluted to 0.2 mg/ml was mixed with 3 ml of 0.2 mM methanolic DPPH solution. After 20 min in the dark at room temperature, the absorbance of the mixture was measured at 517 nm using a 7315 UV spectrophotometer (JENWAY, Staffordshire, United Kingdom). Electron-donating ability (EDA) was calculated as described as follows:

(1) $$\begin{array}{c} \text{EDA}\left(\%\right)=\frac{{\text{ABS}}_{\text{blank}}-{\text{ABS}}_{\text{sample}}}{{\text{ABS}}_{\text{blank}}}\times 100, \end{array}$$

### 2.3. Cell Culture

hDPCs were obtained from Cell Engineering for Origin (CEFO BIO, Seoul, Korea). The cells were grown in Dulbecco's modified Eagle's medium (DMEM) with 1% antibiotics and 10% fetal bovine serum (FBS) at 37*°*C in a 5% CO_2_ humidified atmosphere.

### 2.4. CCK-8 Assay

A CCK-8 assay was used to determine cell proliferation. The hDPCs were seeded at a 3 × 10^3^ cells/well density in 96-well microplates for 24 h at 5% CO_2_ and 37*°*C. The NS extract was diluted to 7 different concentrations (3.91, 7.81, 15.63, 31.52, 62.5, 125, and 250 ppm), and the cells were allowed to grow in 10 *μ*l of each NS concentration for 24 h, as described in several previous hair loss studies [10, 23, 24]. DMEM medium, minoxidil, and triton X-100 (Sigma) were used as a negative control (NC), positive control (PC), and blank, respectively. The plates were treated for 24 h at 5% CO_2_ and 37*°*C. Then, 10 *μ*l of CCK-8 solution (Sigma Aldrich, St. Louis, Mo., USA) was added to each well, and the cells were incubated for 4 h at 37*°*C. The absorbance was measured at 450 nm (test wavelength) and 650 nm (reference wavelength) with a microplate spectrophotometer (Epoch Multi-Volume Spectrophotometer System, BioTek, VT, USA). The measured absorbances were used to determine cell proliferation as follows:

(2) $$\begin{array}{c} \text{cell proliferation}\left(\%\right)=\frac{{\left({\text{ABS}}_{\text{sample}}-{\text{ABS}}_{\text{blank}}\right)}_{450\text{nm}}-{\left({\text{ABS}}_{\text{sample}}-{\text{ABS}}_{\text{blank}}\right)}_{650\text{nm}}}{{\left({\text{ABS}}_{\text{control}}-{\text{ABS}}_{\text{blank}}\right)}_{450\text{nm}}-{\left({\text{ABS}}_{\text{control}}-{\text{ABS}}_{\text{blank}}\right)}_{650\text{nm}}}\times 100. \end{array}$$

### 2.5. Scratch Migration Assay

A scratch migration assay was conducted as described in a previous study [25]. The hDPCs were seeded at a 4 × 10^4^ cell/well density in 6-well plates and grown to 90% confluence in DMEM containing 10% FBS and 1% penicillin. The cells were scratched in a straight line with a 200 *μ*l pipette tip. Cell debris was removed by washing with Dulbecco's phosphate-buffered saline (DPBS). The hDPCs were treated with DMEM (NC, negative control), minoxidil 10 *μ*M (PC, positive control), and 15.63, 31.25, 62.5, and 125 ppm of NS extracts. After incubation for 24 h, the widths of the scratches were photographed using an optical microscope (Leica DM500, Wetzlar, Germany) (40x magnification).

### 2.6. Experimental Animals

Male C57BL/6 mice (4-week-old) purchased from Dae-Han Biolink Co. (Eumsung, Chungbuk, Korea) were allowed to acclimate for 3 weeks. The mice were housed in cages at 23 ± 1*°*C and 55 ± 5% humidity, with a 12 h light and dark cycle and free access to food and water. All experimental protocols followed the guidelines established for the management and handling of laboratory animals and were approved by the Institutional Animal Ethics Committee of Chung-Ang University, Korea (registration no. 201900112). Twenty-one mice (*n* = 7/group) were randomized into the DMSO (NC), Nelumbinis Semen (NS) extract, and 3% minoxidil (PC) treatment groups. The backs of the mice were shaved with animal clippers at 7 weeks of age. Afterward, 200 *μ*l DMSO, 200 *μ*l of NS extract (1,000 ppm), or 3% minoxidil was administered daily on the dorsal skin for 3 weeks using a brush, as described in previous studies [23, 24, 26]. At 21 days, the mice were anesthetized via intraperitoneal injection of 1.2% avertin to obtain dorsal skin samples.

### 2.7. Evaluation of Hair Growth Score and Relative Area for Each Score

To compare the hair growth effect for each group, dorsal skin photographs of the mice were obtained using a digital camera on the 14th day after hair removal. The hair growth index was quantified using the ImageJ program (Broken Symmetry Software, Bethesda, USA). Hair scores were divided into 4 levels according to the differences in the dorsal skin color of the mice from 0 to 3. A score of 0 denoted no hair growth with pink skin color, 1 point indicated that the skin color was gray, 2 points indicated the appearance of some hair growth with (dark gray skin color), and 3 points denoted hair that was completely grown (black skin color). The hair growth index was calculated using equation (3) by measuring the ratio of the area of hair growth to the total area, and the sum of the area ratio multiplied by the score was averaged for each group [27]. Thus, the minimum value of the hair growth index was 0 and the maximum value was 3:

(3) $$\begin{array}{c} \text{hair growth score}=\frac{\left\{\left(\text{area of pink}\right)\times 0+\left(\text{area of gray}\right)\times 1+\left(\text{area of dark gray}\right)\times 2+\left(\text{area of black}\right)\times 3\right\}}{\text{total area}}. \end{array}$$

### 2.8. Histological Analysis

To observe the histological change after the application of NS extract, the tissues were stained with hematoxylin & eosin (H&E). Mice dorsal skin tissues were fixed with 10% neutral formalin and embedded in paraffin blocks using a Tissue Tek Auto TEC Automated Embedder (Sakura, San Francisco, CA, USA). All 4 *μ*m of sections were stained with H&E using a slide stainer (Shandon Linistain GLX, Shandon Inc., Pittsburgh, PA, USA). A veterinary pathologist then examined the histopathologic lesions in the tissues.

### 2.9. Real-Time PCR for Cytokine Analysis of Mice Tissue

Our study assessed the relative expression levels of cytokines involved in hair growth. After 3 weeks of administering the different treatments tested herein, the mice were sacrificed and their back tissues were collected. Then, the tissues were ground in lysis buffer using a homogenizer. mRNA was extracted using the RNeasy Mini Kit (QLAGEN, Germany). Once the total mRNA was extracted from the dorsal skin tissue of each C57BL/6 mice, cDNA was synthesized via reverse transcription. Real-time polymerase chain reaction (RT-PCR) was conducted using a Piko-real 96 real-time PCR system (Thermo Fisher Scientific Inc.; Waltham, MA, USA; 45 cycles at 95*°*C for 15 sec, 60*°*C for 30 sec, and 72*°*C for 30 sec).  Table 1 summarizes the primer sequences for IGF-1, VEGF, and TGF-*β*1.

### 2.10. Statistical Analysis

Statistical analyses were conducted using SPSS version 25 (SPSS Inc., Chicago, USA). All data are reported as mean ± standard error (SE). Statistical analyses were conducted via one-way analysis of variance (ANOVA), and mean differences were analyzed via Duncan's multiple range test. Statistical significance was defined as *p* < 0.05.

## 3. Results

### 3.1. Antioxidant Activity of NS Extract

The antioxidant activity of NS extract is shown in Table 2. The total polyphenol content of NS was 49.52 ± 4.10 mg GAE/g extract weight, and the total flavonoid content of NS was 82.80 ± 4.01 mg NE/g extract weight. The EDA of NS was 78.81%.

### 3.2. Effect of NS Extract on Human Dermal Papilla Cell Proliferation

**Figure 1:**
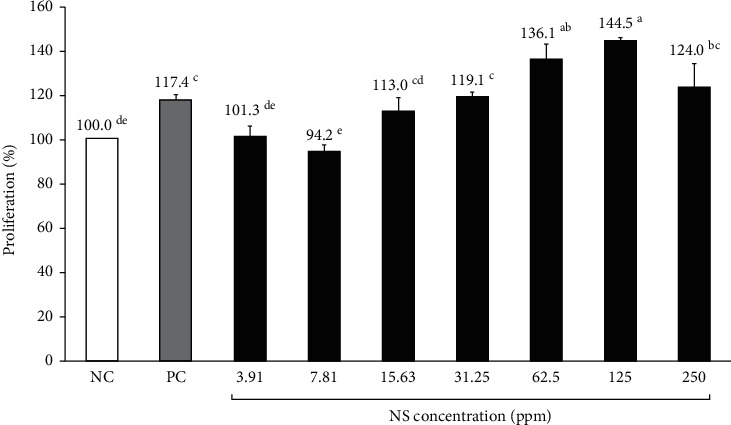
Proliferation of hDPC treated with various concentrations of Nelumbinis Semen extract. Effect of NS extract on hDPC proliferation, as determined by the CCK-8 assay. NC: negative control (DMEM medium), PC: positive control (10 *μ*M minoxidil). The hDPCs were treated with various concentrations of NS extract (3.91, 7.81, 15.63, 31.25, 62.5, 125, and 250 ppm). ^a-d^Values with different superscripts were found to be significantly different (*p* < 0.05), as determined by Duncan's multiple range test.

Figure 1 shows the results of hDPC proliferation at various NS extract concentrations. hDPCs were treated with a range of NS extract concentrations (3.91, 7.81, 15.63, 31.25, 62.5, 125, and 250 ppm), after which cell proliferation rates were measured following incubation for 24 hours. The 3.91 ppm and 7.81 ppm NS extract exposure groups exhibited similar cell proliferation rates to the NC group (101.3% and 94.2%, respectively) when the cell proliferation rate of the NC group was adjusted to 100%. Except for the 15.63 ppm NS treatment, all NS extracts (31.25 ppm, 62.5 ppm, 125 ppm, and 250 ppm) resulted in higher cell proliferation rates than the PC group (117.4%), reaching 119.1%, 136.1%, 144.5%, and 124.0%, respectively. Particularly, the 62.5 ppm and 125 ppm NS extracts exhibited significantly higher cell proliferation rates than the PC group.

### 3.3. Effect of NS Extract on Human Dermal Papilla Cell Migration

**Figure 2:**
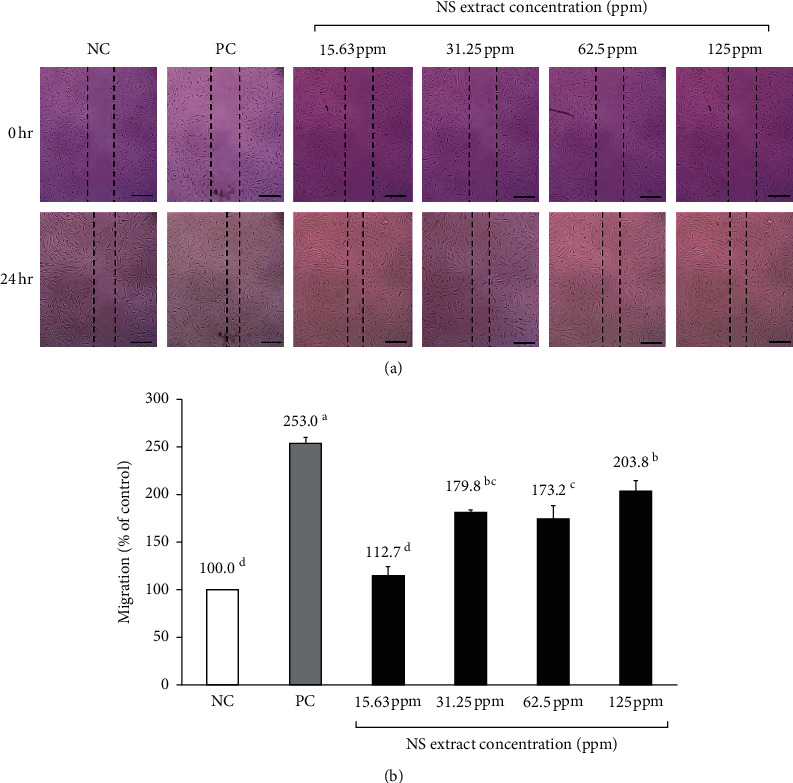
Migration of cells treated with various concentrations of Nelumbinis Semen extract. Effect of Nelumbinis Semen extracts on hDPC migration, as determined by the scratch assay. (a) Scratch line of each group. (b) Degree of migration of the scratch line. NC: negative control (DMEM media), PC: positive control (10 *μ*M minoxidil). ^a-d^Values with different superscripts were found to be significantly different (*p* < 0.05), as determined by Duncan's multiple range test.

The effect of NS extract on hDPCs migration was measured using a scratch migration assay, and the alteration of the scratch line width was converted into a percentage (Figure 2). All NS extract groups from 15.63 to 125 ppm increased the migration of hDPCs compared to the NC group. The migration of hDPCs was the most active at 125 ppm NS (203.8 ± 11.2%) after the PC group (253.0 ± 7.0%), which was significantly higher than the NC group. Treatment with 31.25 ppm and 62.5 ppm of NS resulted in 179.8 ± 14.6% and 173.2 ± 3.7% migration rates, respectively, and thus was found to promote hDPC migration compared with the NC group (*p* < 0.05). Moreover, the 15.63 ppm NS extract also rendered a higher migration rate (112.7 ± 10.5%) than the NC group; however, this difference was not significant. Overall, the migration of hDPCs increased as the concentration of NS extract increased, which was consistent with the cell proliferation rate of CCK-8.

### 3.4. Visual Observation of Hair Growth

**Figure 3:**
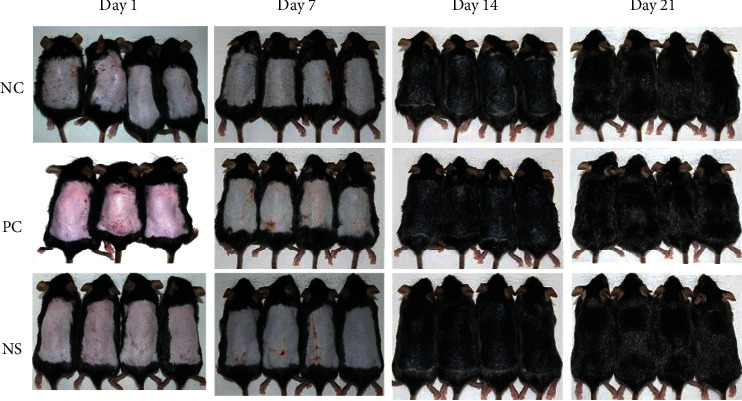
Effect of Nelumbinis Semen extract on anagen phase induction in 7-week-old C57BL/6 mice upon depilation. After depilation, the dorsal skin was treated with DMSO (vehicle), 3% minoxidil, or NS extract (1,000 *μ*g/mL dissolved in DMSO) and photographed at 1, 7, 14, and 21 days (NC: dimethyl sulfoxide (DMSO); PC: 3% minoxidil; NS: Nelumbinis Semen extract with DMSO).

Upon visually confirming the hair growth pattern on the dorsal skin of each mice group after hair removal, the dorsal skin was partially pale gray in all groups on the 7th day, but no significant differences between groups were observed. On the 14th day, hair growth was observed in all mice; however, both the PC and NS extract groups had a darker dorsal skin color than the NC group and exhibited more hair growth. The NS extract group was also found to have a wider area where the hair was completely grown compared to the PC group. After 21 days, hair growth was complete in all groups (Figure 3).

### 3.5. Effects of NS Extract on Hair Growth in C57BL/6 Mice

**Figure 4:**
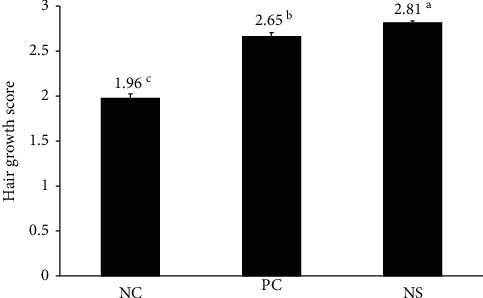
Effect of Nelumbinis Semen extract on hair growth score in C57BL/6 mice. Hair growth scores were calculated at 14 days. Hair growth was significantly promoted by the administration of NS extract compared to the control groups (*p* < 0.05). Differences were calculated by measuring the size of the complete black area (black skin) using ImageJ. ^a-c^Values with different superscripts were found to be significantly different (*p* < 0.05), as determined by Duncan's multiple range test (*n* = 7/group). NC: dimethyl sulfoxide (DMSO); PC: 3% minoxidil; NS: Nelumbinis Semen extract with DMSO.

After calculating the hair growth of all groups on day 14 using the ImageJ program (Broken Symmetry Software, Bethesda, USA) (Figure 4), the PC group (score: 2.65 ± 0.05) and the NS extract group (score: 2.80 ± 0.03) showed a significantly higher hair growth index than the NC group (score: 1.96 ± 0.65). Particularly, the NS extract group was found to have a significantly higher hair growth index than the PC group. The PC group exhibited an approximately 35% higher hair growth effect than the NC treatment, whereas the NS extract group exhibited a 43% higher hair growth effect than the NC treatment.

**Figure 5:**
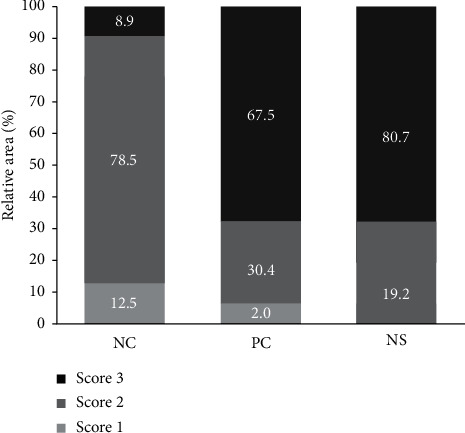
Effect of Nelumbinis Semen extract on relative hair growth area in C57BL/6 mice. The relative area for each score was calculated at 14 days. Differences were calculated by measuring the size of the complete black area (black skin) using the ImageJ program. Values are the percentages for each score (*n* = 7/group). NC: dimethyl sulfoxide (DMSO); PC: 3% minoxidil; NS: Nelumbinis Semen extract with DMSO.

Additionally, after measuring the area of the back of each mouse according to the above-described 0 to 3 hair growth score (Figure 5), the dorsal area of the NC group covered with a score of 2 (78.5%) was the highest (*p* < 0.05), whereas the NS extract group (19.2%) and PC group (30.4%) showed no significant differences. In hair growth scores of 3, where hair growth was complete, the hair growth area was the highest in the NS extract group (80.7%), followed by the PC group (67.5%) and the NC group (8.9%), with the NS extract group being significantly higher than the other groups.

### 3.6. Effects of NS Extract on Hair Morphology

**Figure 6:**
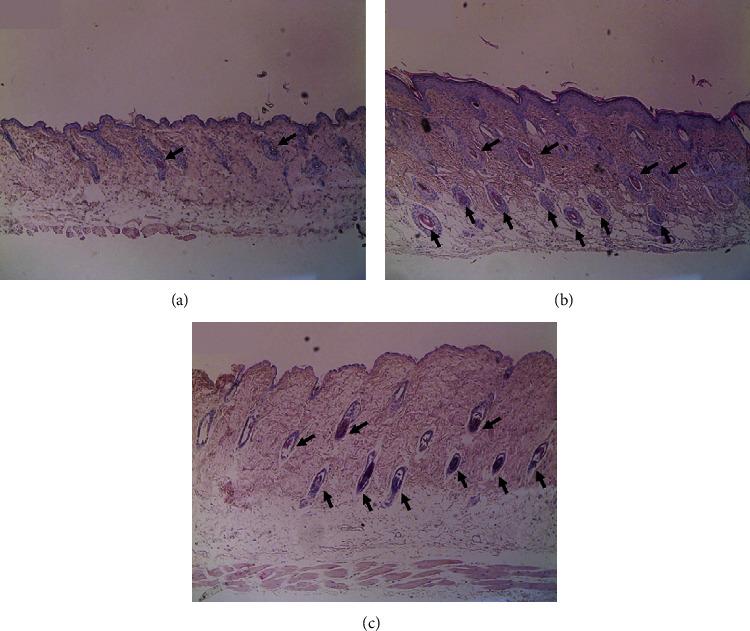
Histological changes in the dorsal skin of C57BL/6 mice after 21 days. (a) NC: dimethyl sulfoxide (DMSO), (b) PC: 3% minoxidil, and (c) NS: Nelumbinis Semen extract with DMSO (H&E staining, 100x magnification).

Histological analyses of dorsal skin tissues were performed to investigate the effect of NS extract on the development of hair follicles and hair roots. After 3 weeks of topical treatment, representative longitudinal sections of dorsal skin tissue from mice were analyzed after staining with hematoxylin & eosin (Figure 6). The hair follicles of the NC group were mainly located in the dermis, and the development of hair follicles was weak. This differed from the PC group, where it was confirmed that hair follicles were located in the deep subcutis and that hair root development was accelerated. The hair follicles of the NS group were located in subcutaneous tissue (i.e., deeper than in the NC group) and appeared larger, resulting in the development of hair roots.

### 3.7. Effect of NS Extract on Cytokine Expression in Mice Dorsal Tissues

**Figure 7:**
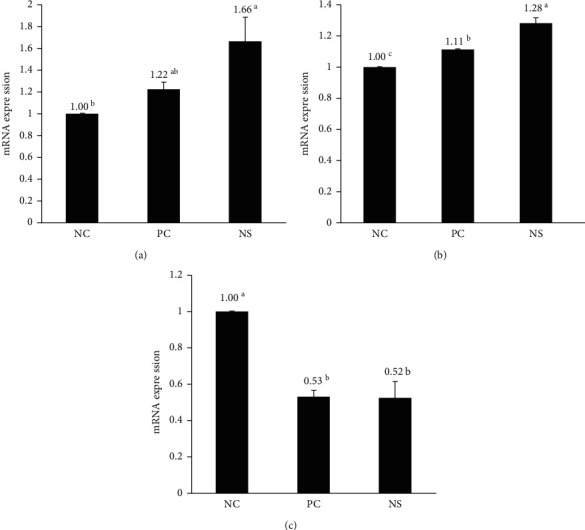
Effect of Nelumbinis Semen extract on the relative mRNA expression of growth factors. Relative expression of growth factors (VEGF, IGF-1, and TGF-*β*1) was analyzed via real-time PCR. NC: negative control (DMSO), PC: positive control (3% minoxidil), NS: Nelumbinis Semen extract. ^a-c^Values with different superscripts were found to be significantly different (*p* < 0.05), as determined by Duncan's multiple range test (*n* = 7/group).

To confirm the hair-growth-promoting effect of NS extract, we compared the mRNA expression levels of hair growth factors using RT-PCR (Figure 7). All gene expression levels were reported relative to the NC group, which was adjusted to 1.0 to calculate the results. The mRNA expression of VEGF, a factor that improves blood circulation by promoting the growth of the vascular endothelium and enhancing hair growth and differentiation of hair root cells, was the highest in the NS extract group (1.66 ± 0.22), which was statistically different from the NC group (*p* < 0.05). Additionally, the expression level of VEGF mRNA in the NS extract group was approximately 1.4 times higher than that in the PC group. IGF-1 is an important growth factor that regulates hair growth by promoting the proliferation of epithelial cells and increasing the length of hair follicle tissue. In this study, the mRNA expression level of IGF-1 was the highest in the NS extract group (1.28 ± 0.04), followed by the PC group (1.11 ± 0.00) and the NC group (1.00 ± 0) (*p* < 0.05). TGF-*β*1 accelerates the transition of the hair growth cycle from the anagen to the catagen phase and suppresses the proliferation and spread of hair follicles. The mRNA expression level of TGF-*β*1 was significantly lower in the PC group (0.53 ± 0.04) and the NS extract group (0.52 ± 0.09) than in the NC group (1.00 ± 0). There was no significant difference between the PC group and the NS group.

## 4. Discussion

In addition to genetic factors, alopecia is caused by excessive stress, malnutrition, hormonal imbalances, and aging and is accompanied by a reduction in hair follicle size and anagen follicles [28–30]. Currently, many studies have been conducted using natural materials, such as herbal medicines and biopharmaceuticals, to identify therapeutic compounds capable of promoting hair growth and reducing hair loss [31–34]. While the activity of glutathione and SOD etc. in the blood of hair loss patients has been found to be significantly low in hair loss patients, the levels of thiobarbituric acid reactive substances (TBARS) are significantly higher than those of patients without hair loss [34]. The resulting oxidative stress has been reported as the likely cause of hair loss [19]. However, these sources of oxidative stress can be reduced via the activity of natural antioxidant substances such as polyphenols, flavonoids, and phenol compounds, which are abundant in natural plant materials. Their ability to reduce oxidative stress derives from their capacity to remove free radicals and balance the reactive oxygen species metabolism [20]. NS is also thought to improve oxidative stress in the scalp of alopecia patients due to its high total polyphenol and flavonoid content, as well as its high DPPH electron radical scavenging activity [14, 15]. Dlova and Ollengo [35] reported that anthraquinone, flavonoids, tannin, saponin, chrysophanol, aloe-emodin, aloesin, and aloenin, among others, prevent hair loss and promote hair growth. Among these substances, anthraquinone, flavonoids, tannins, and saponins are the main active ingredients in NS [14]. Despite this, very few studies have assessed the hair growth and hair loss reduction potential of NS extract. Therefore, our study sought to investigate the hair-growth-promoting properties of NS extract through *in vitro* experiments using hDPCs and *in vivo* experiments using C57BL/6 mice.

According to previous studies, antioxidant substances suppress hair loss-promoting cytokine activity, thereby promoting hair growth [34, 36]. A study by Kim et al. [37] reported that the average flavonoid content of 40 native plants was 70 mg NE/g. The flavonoid content of NS extract is approximately 1.2 times higher. Additionally, this study found that the EDA of NS extract was approximately 1.1 times higher than the average EDA value of 74% for native plants. Asha et al. [38] found that *Rhizophora apiculata* root extract, an extract rich in flavonoids and polyphenolic compounds, effectively reduced oxidative stress in rat brains. Additionally, preclinical studies using natural extracts found that the antioxidant activity of natural substances reduces not only oxidative damage but also hair loss by promoting the expression of hair growth factors and participating in cytokine-mediated inflammation [39]. As expected, this study found that NS extract possessed a strong antioxidant capacity due to its high flavonoid content and EDA, which may reduce the oxidative damage that causes hair loss.

hDPCs make up the hair follicles and are connected to blood vessels, which supply them with nutrients, hormones, and other secreted physiological substances, such as hair growth factors. These secretory mediators affect the surrounding hair follicle cells and tissues and play an important role in hair growth and detachment [19, 40]. Driskell et al. [41] reported that hair growth could be enhanced by promoting the proliferation of hDPCs and human epidermal keratinocyte cells (HaCaT). The results of this study showed that cell proliferation and migration tend to increase as the concentration of NS extract increases. Notably, 31.25 ppm of NS extract (i.e., a relatively low concentration) resulted in a cell proliferation rate that was higher than that of minoxidil, the positive control. Previous studies have reported that plant extracts or compounds can be deemed nontoxic if cell proliferation rates exceed 85% upon their administration [23]. Both *Geranium sibiricum* extract and *Miscanthus sinensis* var*. purpurascens* extract led to cell proliferation rates below 85% at 156.3 and 62.5 ppm [24], respectively, and were, therefore, considered cytotoxic. Similarly, a *Salvia plebeia* extract substantially reduced cell proliferation rates at 125 ppm [26]. In other words, most plant extracts have been found to be toxic at high concentrations; however, the NS extracts in this study were found to be nontoxic at concentrations as high as 250 ppm, and cell proliferation rates exceeded 85% at all tested concentrations (3.91–250 ppm). Therefore, NS extracts were deemed nontoxic and likely constitute a safe hair loss treatment.

In this study, C57BL/6 mice were selected to analyze hair growth promotion and hair loss prevention. All experiments were initiated when the test mice were 7 weeks old, which is when the hair cycle enters the telogen phase. C57BL/6 mice exhibit spontaneous hair loss and are, therefore, used as representative experimental animals in alopecia research because melanocytes exist only in their hair follicles and melanin synthesis is consistent with the hair growth cycle, which can be assessed simply by observing skin color [42]. Upon measuring the hair growth index of all groups on the 14th day, it was confirmed that the NS extract group had a higher hair growth index than the PC group (*p* < 0.05). Moreover, the PC group in our study achieved a 68% hair recovery, whereas the NS extract group reached 81% within the same time. Another study reported that the administration of a medicinal herb in C57BL/6 mice resulted in a hair growth index of 1.7 points [27]. Additionally, *Saengbal-eum-II*, a medicinal herb known to possess good hair-growth-promoting effects, had a high hair growth index of 2.28 points [43]. However, NS reached a growth index of 2.81 points, thus substantially exceeding the effects of the aforementioned herb. Therefore, these results highlight the excellent efficacy of NS extracts to promote hair growth and preventing hair loss.

HFs are composed of several layers of cells, the hair outer root sheath (ORS) and inner root sheath (IRS), hair cells, and papillary cells (DPCs), and play an important role in physiological tissue regeneration. Stem cells in the bulge region of HFs and DPCs play key roles in the regulation of successive hair cycles. These HFs undergo the anagen, catagen, and telogen phases [1, 2]. Most telogen hair follicles are located in the dermis, whereas anagen hair follicles are found only in the deep subcutis [44]. Our study also confirmed that the hair follicles and hair bulbs of the NS extract group were larger than those of the NC group. Additionally, the hair follicles of the NS extract group were found to be located in deeper subcutaneous tissue than those of the NC group. These results suggest that NS extracts promote hair growth by accelerating the telogen to anagen transition of hair follicles.

The dermal papilla cells derived from mesenchymal cells are affected by specific cytokines, and the affected dermal papilla cells regulate the growth or degeneration of hair by releasing growth factors or inhibitors of the epithelial cells of the hair follicles. Overexpression of VEGF in the outer root sheath reportedly promotes hair growth by promoting the development of blood vessels around the hair follicles [45]. In this study, VEGF mRNA expression was higher in the NS extract group than in the minoxidil group. These results are similar to those of a study that evaluated the effect of *Geranium sibiricum* extract on hair growth [23]. The mRNA expression level of IGF-1 was significantly higher in the NS extract group than in the PC group. Transgenic mice that overexpressed IGF-1 in their skin exhibited faster HF growth than the control group. This finding is supported by a study that found that the hair of patients with Laron syndrome, an IGF-1 deficiency, grows sparsely and frequently falls out in the frontal region [45]. Previous studies have shown that when the expression of TGF-*β*1 is increased, hair follicle proliferation is not completed and hair follicle regeneration is reduced, resulting in thin, short hair [46]. This study found that the mRNA expression of TGF-*β*1 was significantly lower in the NS extract group than in the NC group, which is consistent with previous studies on the hair-growth-promoting effects of *Salvia plebeian* extracts and *Laminaria japonica* extracts [26, 47]. Based on these results, we confirmed that NS extract improves hair growth by increasing the expression of hair growth-promoting cytokines such as VEGF and IGF-1, while also reducing the expression of TGF-*β*1, which inhibits hair growth.

## 5. Conclusions

Our findings confirmed that NS extract promotes hair growth by enhancing hDPC proliferation and migration *in vitro*. Additionally, the hair growth index and hair growth area of C57BL/6 mice topically treated with NS extract for 3 weeks increased much more than in minoxidil-treated mice. Moreover, the number and location of hair follicles considered to be evidence of anagen induction were similar in the NS extract group compared to the minoxidil group. NS extract treatment also increased the mRNA expression of VEGF and IGF-1, which are cytokines involved in hair growth, and decreased the expression of TGF-*β*1, a hair growth inhibitor. Therefore, NS extract is a promising new hair loss treatment derived from natural substances that may be used to promote hair growth and prevent hair loss.

## Figures and Tables

**Table 1:** Primer pairs used for quantitative real-time RT-PCR.

Target genes	Primer	Sequence
IGF-1	Forward	5′-TGCTCTTCAGTTCGTGTG-3′
	Reverse	5′-ACATCTCCAGTCTCCTCAG-3′
VEGF	Forward	5′-TCTTCAAGCCATCCTGTGTG-3′
	Reverse	5′-GCGAGTCTGTGTTTTTGCAG-3′
TGF-*β*1	Forward	5′-GGCGGTGCTCGCTTTGTAC-3′
	Reverse	5′-TCCCGAATGTCTGA CGTATTGA-3′
GAPDH	Forward	5′-GGGAAGCCCATCACCATCT-3′
	Reverse	5′-CGGCCTCACCCCATTTG-3′

**Table 2:** Total polyphenol, flavonoid content, and electron-donating ability of Nelumbinis Semen extract.

	Total polyphenols (mg GAE^1^/g)	Total flavonoids (mg NE^2^/g)	EDA^3^ (%)
Nelumbinis Semen	49.52 ± 4.10	82.80 ± 4.01	78.81

All data are reported as mean ± standard error (SE). ^1^GAE: gallic acid equivalent. ^2^NE: naringin equivalent. ^3^EDA: electron-donating ability.

## Data Availability

The datasets used to support the findings of this study are available from the corresponding author upon request.

## Abbreviations

AA: Alopecia areata
AGA: Androgenetic alopecia
DHT: Dihydrotestosterone
DMEM: Dulbecco's modified Eagle's medium
DPBS: Dulbecco's phosphate-buffered saline
EDA: Electron-donating ability
FBS: Fetal bovine serum
FPHL: Female pattern hair loss
GAE: Gallic acid equivalent
H&E: Hematoxylin and eosin
HaCaT: Human epidermal keratinocyte cells
hDPCs: Human dermal papilla cells
HFs: Hair follicles
IGF-1: Insulin-like growth factor-1
IRS: Inner root sheath
NE: Naringin equivalent
NS: Nelumbinis Semen
ORS: Outer root sheath
RT-PCR: Real-time polymerase chain reaction
T: Testosterone
TBARS: Thiobarbituric acid reactive substances
TGF-*β*1: Transforming growth factor-*β*1
VEGF: Vascular endothelial growth factor.
